# Reemergence of Endemic Chikungunya, Malaysia

**DOI:** 10.3201/eid1301.060617

**Published:** 2007-01

**Authors:** Sazaly AbuBakar, I-Ching Sam, Pooi-Fong Wong, NorAziyah MatRahim, Poh-Sim Hooi, Nuruliza Roslan

**Affiliations:** * University of Malaya, Kuala Lumpur, Malaysia

**Keywords:** Chikungunya virus, Malaysia, dispatch

## Abstract

Chikungunya virus infection recently reemerged in Malaysia after 7 years of nondetection. Genomic sequences of recovered isolates were highly similar to those of Malaysian isolates from the 1998 outbreak. The reemergence of the infection is not part of the epidemics in other Indian Ocean countries but raises the possibility that chikungunya virus is endemic in Malaysia.

Chikungunya, a mosquitoborne disease first described in Tanzania (formerly Tanganyika) in eastern Africa in 1952, is caused by chikungunya virus (CHIKV), an alphavirus belonging to the *Togaviridae* family. The disease occurs in Africa and various parts of Asia and is endemic in several southeast Asian countries, including Thailand, Indonesia, and the Philippines. Only 1 known outbreak has occurred in Malaysia, in 1998–1999 when >51 persons in Port Klang were infected (*1*).

From March through April 2006, an outbreak of CHIKV infection was reported in Bagan Panchor (4°31′N, 100°37′E), an isolated coastal town 50 km west of Ipoh, the state capital of Perak, in northwest Malaysia. At least 200 villagers were infected, with no deaths reported. This was the second known outbreak in Malaysia, 7 years after the previous one. This reemergence coincided with reports of ongoing epidemics of CHIKV infection in India and almost all the island nations of the Indian Ocean, with >200,000 cases in the French island of Reunion alone since February 2005 (*2*).

Why and how the recent infection reappeared in Malaysia remains unknown. The apparent absence of CHIKV for 7 years may be due to failure to detect low-level, continued transmission in humans, particularly because the symptoms may be mistaken for dengue fever. Alternatively, this outbreak could have originated from a viremic traveler from an endemic country (such as neighboring Thailand or Indonesia), but proximity of Malaysia to the Indian Ocean raises the possibility of an extension of the epidemic, with Malaysia being the furthest point yet of the expanding epidemic frontline.

## The Study

We received serum samples from 11 patients who had symptoms typical of CHIKV infection (Table). Samples were injected into Vero and C6/36 mosquito cells. Indirect immunofluorescence assays for immunoglobulin M (IgM) and IgG were performed using the patients’ sera and CHIKV-infected cells fixed onto glass slides, as previously described (*1*). A CHIKV isolate (SM287) reported previously (*3*) was used to prepare the slides as a positive control for subsequent studies. Serum samples from patients who did not have symptoms of chikungunya, including patients with dengue fever, were used as negative controls. Nucleic acid amplification was performed using RNA extracted directly from the patients’ sera or from cell cultures (Table). At least 3 different primer pairs specific for envelope glycoprotein E1 (E1), glycoprotein E2 (E2), and nonstructural protein 1 (nsP1) genes of CHIKV were used (*4*,*5*). Confirmation of the amplified DNA fragments was done by DNA sequencing. Phylogenetic relationships were examined using the E1, E2, and nsP1 gene sequences of the isolates and all other available CHIKV sequences obtained from GenBank or the previous studies (Appendix Table). Sequences were aligned and phylogenetic trees were drawn as previously described (*6*).

**Table:** Identification of virus by PCR amplification and serologic analysis*

Patient		Chikungunya						Dengue fever	
Age (y)	Sex	PCR†			Serology		Culture	PCR‡	Serology
		E1	E2	nsP1	IgM	IgG			IgM
6	M	+	+	+	–	−	+§	−	−
34	M	+	+	+	–	−	+¶	−	−
40	M	+	+	+	–	−	+#	−	−
26	F	+	+	+	–	−	+**	ND	−
62	M	+	+	+	–	−	−††	ND	−
		(day 5 after onset)							
		–	–	–	+	+	ND	ND	ND
		(day 15 after onset)							

*IgM, immunoglobulin M; IgG, immunoglobulin G; +, positive; −, negative; ND, not determined. †PCR amplifications were performed for detection of envelope glycoprotein E1 (E1), glycoprotein E2 (E2), and nonstructural protein 1 (nsP1) genes of chikungunya virus. ‡Multiplex PCR amplifications were performed for detection of dengue virus type 1−4. §Isolate MY/0306/BP37348. ¶Isolate MY/0306/BP37350. #Isolate MY/0306/BP37352. **Isolate MY/0406/BP37437. ††Isolate MY/0306/BP34198.

CHIKV infection was confirmed in 5 of 11 patients. CHIKV sequences were amplified directly from serum samples from 5 patients in the acute phase of disease. Of these, 4 CHIKV isolates were eventually cultured. IgM and IgG were detected in serum samples from 3 other patients in the convalescent phase (data not shown). In 1 patient, CHIKV sequences were amplified from serum samples obtained as late as 9 days after onset of symptoms (data not shown). The PCR amplification method, thus, could be useful for early detection of CHIKV infection in suspected outbreak situations.

The genomic sequence of the E1, E2, and nsP1 genes in the CHIKV isolates shared high similarity (>90%) to all the known CHIKV except West African CHIKV (≈86% similarity). The sequences were only ≈70% related to O’nyong-nyong virus, the most closely related alphavirus, which is present only in certain parts of Africa. Previous phylogenetic studies showed that CHIKV strains were clustered into 3 distinct groups based on origin from West Africa, Central/East Africa, or Asia (*7*–*13*). Phylogenetic trees drawn using E1 (Figure), E2, and nsP1 (data not shown) gene sequences clustered the recent Malaysian isolates into a group with other known CHIKV Asian isolates. The cluster, however, was distinctly separated (100% bootstrap support) from the African isolates and all the known isolates of the ongoing CHIKV epidemics of the Indian Ocean islands (*7*–*9*,*11*,*13*). This makes it unlikely that the outbreak in Malaysia is part of the ongoing epidemics, despite its proximity to the region and timing of the outbreak. The phylogenetic tree, on the other hand, suggests that the isolates from the current Malaysia outbreak share a common ancestral lineage to the 2 Malaysian isolates recovered in 1998 (*4*; GenBank accession nos. AF394210 and AF394211) but have a slight genetic distance from all other Asian isolates.

**Figure:**
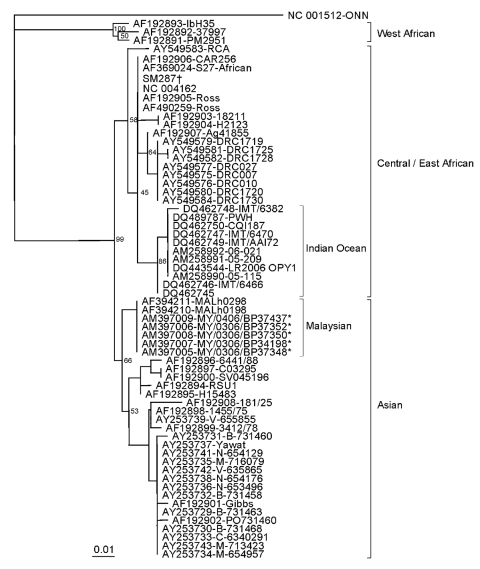
Phylogenetic relationships of chikungunya virus isolates from the Malaysia 2006 outbreak. The neighbor-joining tree was constructed using nucleic acid sequences of the envelope glycoprotein E1 gene, with O’nyong nyong virus (GenBank accession no. NC_001512) as the outgroup virus. * indicates isolates from the Malaysia 2006 outbreak; † indicates Australia SM287. Bootstrap values are shown as percentages derived from 1,000 samplings. The scale reflects the number of nucleotide substitutions per site along the branches.

## Conclusions

On the basis of all available sequences of isolates from the neighboring countries where CHIKV is endemic, Thailand and Indonesia, the outbreak in Malaysia likely did not originate from either of these countries, which means the outbreak could have originated from an endemic CHIKV cycle not previously identified in Malaysia. A serologic survey of human serum samples collected during 1965–1969 in west Malaysia showed neutralizing antibodies to CHIKV among adults, especially those inhabiting the rural northern and eastern states bordering Thailand (*14*). The same authors also reported in an earlier study evidence of CHIKV-neutralizing antibodies in wild monkeys, a pig, and a chicken and suggested that a CHIKV sylvatic transmission cycle involving primates and possibly nonprimates exists in Malaysia. A sylvatic transmission cycle of the virus has been described in Africa and may play a role in the episodic emergence and reemergence of CHIKV infection (*15*). Before 1998, CHIKV had not been isolated from humans or animals in Malaysia, and no clinical disease caused by CHIKV had been reported. However, in the absence of active surveillance since the 1965 study, whether the apparent absence of CHIKV over the years and between the 2 recent outbreaks in Malaysia is due to an unidentified sylvatic transmission cycle or silent transmission among humans cannot be determined. Further investigation is required to examine these possibilities. Understanding this disease in Southeast Asia is critical because CHIKV shares the same mosquito vectors as dengue virus, which is endemic to the region.

Phylogenetic analysis showed that CHIKV from the recent 2006 outbreak in Malaysia is highly similar to isolates from the 1998 outbreak. At the 3 genes examined, the isolates differ from the ongoing Indian Ocean epidemic isolates and known isolates from Thailand and Indonesia. These findings support the possibility that the outbreak originated from an endemic infection in Malaysia.

## Supplementary Material

Appendix Table Chikungunya virus envelope glycoprotein E1 sequences used for the phylogenetic analysis*
