# Supplementary material for: Reemergence of Endemic Chikungunya, Malaysia
Source: Emerg Infect Dis. 2007 Jan;13(1):147–9. doi: 10.3201/eid1301.060617 (PMC2725805; doi:10.3201/eid1301.060617)
Supplement: Appendix Table — Chikungunya virus envelope glycoprotein E1 sequences used for the phylogenetic analysis* [file 06-0617_appT-s1.pdf]

Appendix Table. Chikungunya virus envelope glycoprotein E1 sequences used for the phylogenetic analysis*

Isolate	GenBank accession no.	Location	Date of isolation	Ref
IbH35	AF192893	Nigeria	1964	(7)
37997	AF192892	Senegal	1983	(7)
PM2951	AF192891	Senegal	1966	(7)
RCA	AY549583	DRC	1996	(8)
18211	AF192903	SA	1976	(7)
H2123	AF192904	SA	1976	(7)
SM287	NA	Australia	1990	(3)
S27	NC_004162	Tanzania	NA	(12)
S27-African prototype	AF369024	Tanzania	NA	(12)
CAR256	AF192906	CAR	NA	(7)
Ross	AF192905	Tanzania	1953	(7)
Ross	AF490259	Tanzania	NA	†
UG Ag41855	AF192907	Uganda	1982	(7)
DRC1730	AY549584	DRC	1996	(8)
DRC1720	AY549580	DRC	2000	(8)
DRC027	AY549577	DRC	2000	(8)
DRC007	AY549575	DRC	2000	(8)
DRC010	AY549576	DRC	2000	(8)
DRC1725	AY549581	DRC	2000	(8)
DRC1728	AY549582	DRC	2000	(8)
IMT/6470	DQ462747	Réunion	2006	(9)
IMT/6382	DQ462748	Réunion	2006	(9)
LR2006_OPY1	DQ443544	Réunion	2006	(13)
PWH	DQ489787	Hong Kong	2006	†
IMT/AAI72	DQ462749	Réunion	2006	(9)
CQI187	DQ462750	Réunion	2006	(9)
IMT/6466	DQ462746	Réunion	2006	(9)

05-115	AM258990	Réunion	2005	(11)
05-209	AM258991	Réunion	2005	(11)
06-021	AM258992	Réunion	2006	(11)
MALh0198	AF394210	Malaysia	1998	(4)
MALh0298	AF394211	Malaysia	1998	(4)
MY/0306/BP37348	AM397005	Malaysia	2006	‡
MY/0306/BP37350	AM397008	Malaysia	2006	‡
MY/0306/BP37352	AM397006	Malaysia	2006	‡
MY/0406/BP37437	AM397009	Malaysia	2006	‡
MY/0306/BP34198	AM397007	Malaysia	2006	‡
6441/88	AF192896	Thailand	1988	(7)
C03295	AF192897	Thailand	1995	(7)
SV0451/96	AF192900	Thailand	1996	(7)
RSU1	AF192894	Indonesia	1985	(7)
H15483	AF192895	The Philippines	1985	(7)
181/25	AF192908	Thailand	1962	(7)
3412/78	AF192899	Thailand	1978	(7)
V-655855	AY253739	India	NA	(10)
1455/75	AF192898	Thailand	1975	(7)
Gibbs	AF192901	India	1963	(7)
B-731460	AY253731	India	NA	(10)
N-654176	AY253738	India	NA	(10)
N-653496	AY253736	India	NA	(10)
B-731463	AY253729	India	NA	(10)
N-654129	AY253741	India	NA	(10)
Yawat	AY253737	India	NA	(10)
M-713423	AY253743	India	NA	(10)
V-635865	AY253742	India	1965	(10)
M-716079	AY253735	India	NA	(10)
PO731460	AF192902	India	1973	(7)
M-654957	AY253734	India	NA	(10)

C-6340291	AY253733	India	NA	(10)
B-731468	AY253730	India	NA	(10)
B-731458	AY253732	India	NA	(10)

*NA, not available; DRC, Democratic Republic of Congo; SA, Republic of South Africa; CAR, Central African Region; Réunion, La Réunion Island; PWH, Prince of Wales Hospital.
†Unpub. data.
‡This study.
